# Supplementary material for: HDL-Related Parameters and COVID-19 Mortality: The Importance of HDL Function
Source: Antioxidants (Basel). 2023 Nov 16;12(11):2009. doi: 10.3390/antiox12112009 (PMC10669705; doi:10.3390/antiox12112009)
Supplement: Supplementary file 1 [file antioxidants-12-02009-s001.zip › Supplementary Table S1.pdf]

	parameter			data of this study cohort (all samples)					COVID-19 patients					Non-COVID pneumonia controls				
	Lipoproteins																	
	abbreviation	full name	unit	mean	sd	$Q_{2.5\%}$	$Q_{50\%}$	$Q_{97.5\%}$	mean	sd	$Q_{2.5\%}$	$Q_{50\%}$	$Q_{97.5\%}$	mean	sd	$Q_{2.5\%}$	$Q_{50\%}$	$Q_{97.5\%}$
1	TPTG	total triglycerides	mg/dL	143	79	46	120	347	122	55	43	119	238	157	89	57	124	345
2	TPCH	total cholesterol	mg/dL	180	58	79	177	299	201	55	104	207	298	166	56	79	161	290
3	LDCH	LDL cholesterol	mg/dL	93	43	19	89	185	105	45	28	112	179	84	41	28	83	170
4	HDCH	HDL cholesterol	mg/dL	44	17	18	41	82	51	18	23	52	82	39	14	20	37	78
5	TPA1	total Apo-A1	mg/dL	123	37	60	117	215	140	39	73	143	216	111	31	61	110	184
6	TPA2	total Apo-A2	mg/dL	26	8	12	25	44	28	8	12	28	44	24	7	13	23	37
7	TPAB	total Apo-B100	mg/dL	97	32	41	97	173	96	29	50	100	162	97	34	41	96	157
8	LDHD	LDL-cholesterol/HDL-cholesterol ratio	-/-	2,2	1,0	0,6	2,1	4,3	2,1	0,9	0,6	2,1	3,9	2,3	1,1	0,7	2,1	4,2
9	ABA1	Apo-B100/Apo-A1 ratio	-/-	0,8	0,3	0,3	0,8	1,6	0,7	0,3	0,3	0,7	1,4	0,9	0,4	0,4	0,9	1,6
10	TBPN	total particle number	nmol/L	1760	586	737	1768	3143	1753	532	902	1816	2945	1766	625	748	1750	2861
11	VLPN	VLDL particle number	nmol/L	209	117	32	174	471	169	89	30	145	363	235	127	89	178	461
12	IDPN	IDL particle number	nmol/L	155	78	45	138	332	140	64	58	126	274	166	86	46	142	286
13	LDPN	LDL particle number	nmol/L	1371	492	541	1417	2397	1402	480	565	1462	2413	1351	504	549	1416	2292
14	L1PN	LDL-1 particle number	nmol/L	265	120	89	243	515	271	127	109	239	559	260	117	90	260	474
15	L2PN	LDL-2 particle number	nmol/L	141	86	0	135	342	151	93	38	144	347	134	82	1	125	296
16	L3PN	LDL-3 particle number	nmol/L	194	82	69	189	367	206	88	69	207	363	187	79	70	177	348
17	L4PN	LDL-4 particle number	nmol/L	188	115	0	164	458	198	99	0	193	363	181	124	2	160	448
18	L5PN	LDL-5 particle number	nmol/L	231	143	0	230	572	237	144	0	249	499	227	144	1	228	524
19	L6PN	LDL-6 particle number	nmol/L	332	182	0	298	777	337	194	69	323	729	329	175	60	298	685
20	VLTG	VLDL triglycerides	mg/dL	87	55	17	75	242	71	35	24	66	142	98	63	23	78	240
21	IDTG	IDL triglycerides	mg/dL	11	11	0	7	43	8	7	0	7	24	12	12	0	8	41
22	LDTG	LDL triglycerides	mg/dL	36	15	16	34	69	31	12	17	29	56	39	17	16	37	67
23	HDTG	HDL triglycerides	mg/dL	16	6	6	14	34	15	6	6	14	26	16	7	8	15	34
24	VLCH	VLDL cholesterol	mg/dL	24	15	4	21	70	22	14	3	21	55	25	16	7	20	67
25	IDCH	IDL cholesterol	mg/dL	20	11	1	19	44	19	9	5	19	38	20	12	1	18	43
26	VLFC	VLDL free cholesterol	mg/dL	10	6	2	9	26	9	5	2	9	20	11	6	4	9	26
27	IDFC	IDL free cholesterol	mg/dL	5	3	0	5	13	5	3	1	5	11	6	4	0	5	13
28	LDFC	LDL free cholesterol	mg/dL	33	12	12	32	57	36	13	13	37	57	31	12	12	30	53
29	HDFC	HDL free cholesterol	mg/dL	12	6	2	11	25	15	6	3	15	26	11	5	2	10	22
30	VLPL	VLDL phospholipids	mg/dL	19	11	5	17	47	17	9	5	18	35	21	12	8	16	47
31	IDPL	IDL phospholipids	mg/dL	6	4	0	6	17	6	3	1	6	13	6	5	0	6	17
32	LDPL	LDL phospholipids	mg/dL	58	21	23	59	104	63	21	25	62	101	55	20	26	56	99
33	HDPL	HDL phospholipids	mg/dL	67	21	37	63	124	76	24	40	72	125	60	16	37	60	103
34	HDA1	HDL Apo-A1	mg/dL	115	39	55	111	213	133	42	61	137	214	104	32	55	104	183
35	HDA2	HDL Apo-A2	mg/dL	27	7	13	27	44	29	8	13	29	43	26	7	16	25	37
36	VLAB	VLDL Apo-B100	mg/dL	11	6	2	10	26	9	5	2	8	20	13	7	5	10	25
37	IDAB	IDL Apo-B100	mg/dL	9	4	2	8	18	8	3	3	7	15	9	5	3	8	16
38	LDAB	LDL Apo-B100	mg/dL	75	27	30	78	132	77	26	31	80	133	74	28	30	78	126
39	V1TG	VLDL-1 triglycerides	mg/dL	44	37	4	33	151	33	25	4	24	90	51	42	6	39	150
40	V2TG	VLDL-2 triglycerides	mg/dL	11	7	2	9	34	9	5	3	9	19	12	8	3	10	33
41	V3TG	VLDL-3 triglycerides	mg/dL	13	8	3	11	32	11	6	3	10	22	14	9	3	12	31
42	V4TG	VLDL-4 triglycerides	mg/dL	13	8	3	11	29	11	6	3	11	25	14	9	5	11	28
43	V5TG	VLDL-5 triglycerides	mg/dL	4	2	1	4	8	3	1	1	4	6	4	2	2	4	8
44	V1CH	VLDL-1 cholsesterol	mg/dL	6	6	0	5	22	6	6	0	5	20	7	7	1	4	22
45	V2CH	VLDL-2 cholsesterol	mg/dL	3	2	0	2	10	3	2	0	2	8	3	2	0	2	9
46	V3CH	VLDL-3 cholsesterol	mg/dL	4	3	0	4	13	4	3	1	4	11	4	3	1	4	12
47	V4CH	VLDL-4 cholsesterol	mg/dL	8	4	1	7	17	7	4	2	7	16	8	5	2	7	16
48	V5CH	VLDL-5 cholsesterol	mg/dL	2	1	0	1	4	1	1	0	1	4	2	1	0	1	4
49	V1FC	VLDL-1 free cholsesterol	mg/dL	2	2	0	1	7	1	2	0	1	5	2	2	0	1	7
50	V2FC	VLDL-2 free cholsesterol	mg/dL	2	1	0	1	6	1	1	0	1	4	2	1	0	1	6
51	V3FC	VLDL-3 free cholsesterol	mg/dL	2	2	0	2	7	2	1	0	2	5	2	2	1	2	7
52	V4FC	VLDL-4 free cholsesterol	mg/dL	4	2	0	3	9	3	2	0	3	8	4	3	1	3	8
53	V5FC	VLDL-5 free cholsesterol	mg/dL	1	1	0	0	2	1	1	0	0	2	1	1	0	0	2
54	V1PL	VLDL-1 phospholipids	mg/dL	6	5	1	4	21	5	3	0	4	12	7	5	1	5	20

	parameter			data of this study cohort (all samples)					COVID-19 patients					Non-COVID pneumonia controls				
	Lipoproteins																	
	abbreviation	full name	unit	mean	sd	$Q_{2.5\%}$	$Q_{50\%}$	$Q_{97.5\%}$	mean	sd	$Q_{2.5\%}$	$Q_{50\%}$	$Q_{97.5\%}$	mean	sd	$Q_{2.5\%}$	$Q_{50\%}$	$Q_{97.5\%}$
55	V2PL	VLDL-2 phospholipids	mg/dL	3	2	1	2	8	2	1	1	2	5	3	2	1	2	8
56	V3PL	VLDL-3 phospholipids	mg/dL	4	3	0	3	10	4	2	1	4	8	4	3	1	3	10
57	V4PL	VLDL-4 phospholipids	mg/dL	7	4	1	6	14	6	3	1	6	13	7	4	3	6	14
58	V5PL	VLDL-5 phospholipids	mg/dL	2	1	0	2	4	2	1	0	2	4	2	1	0	2	4
59	L1TG	LDL-1 triglycerides	mg/dL	11	6	3	9	29	9	6	3	8	22	12	7	3	12	26
60	L2TG	LDL-2 triglycerides	mg/dL	4	2	2	4	10	4	2	2	3	8	5	2	2	4	9
61	L3TG	LDL-3 triglycerides	mg/dL	4	1	2	3	7	3	1	2	3	6	4	1	2	4	7
62	L4TG	LDL-4 triglycerides	mg/dL	5	3	2	4	11	4	2	2	4	8	6	3	2	5	11
63	L5TG	LDL-5 triglycerides	mg/dL	5	2	1	4	9	4	2	2	4	8	5	3	1	5	9
64	L6TG	LDL-2 triglycerides	mg/dL	5	2	1	5	10	5	2	2	4	10	6	2	2	6	10
65	L1CH	LDL-1 cholsesterol	mg/dL	21	12	0	20	47	24	13	4	24	51	19	11	0	18	40
66	L2CH	LDL-2 cholsesterol	mg/dL	11	9	0	10	36	13	11	0	12	36	10	8	0	10	28
67	L3CH	LDL-3 cholsesterol	mg/dL	14	9	0	14	35	17	10	0	16	35	13	8	1	12	29
68	L4CH	LDL-4 cholsesterol	mg/dL	12	9	0	11	34	14	9	0	16	28	11	10	0	8	34
69	L5CH	LDL-5 cholsesterol	mg/dL	15	11	0	15	43	16	11	0	18	35	14	11	0	14	32
70	L6CH	LDL-6 cholsesterol	mg/dL	21	13	0	19	54	22	14	2	20	50	20	12	1	19	43
71	L1FC	LDL-1 free cholsesterol	mg/dL	7	3	1	7	16	8	3	3	7	16	7	3	1	6	12
72	L2FC	LDL-2 free cholsesterol	mg/dL	4	3	0	4	11	5	3	0	4	11	4	3	0	3	9
73	L3FC	LDL-3 free cholsesterol	mg/dL	5	3	1	5	11	6	3	1	7	11	5	2	2	5	9
74	L4FC	LDL-4 free cholsesterol	mg/dL	5	3	0	5	11	5	3	1	5	10	4	3	0	4	11
75	L5FC	LDL-5 free cholsesterol	mg/dL	5	3	0	5	13	5	3	0	6	11	5	3	0	4	10
76	L6FC	LDL-6 free cholsesterol	mg/dL	6	4	0	6	16	6	4	1	6	15	6	4	0	5	13
77	L1PL	LDL-1 phospholipids	mg/dL	14	7	2	13	28	15	7	4	13	30	13	6	2	12	25
78	L2PL	LDL-2 phospholipids	mg/dL	7	5	0	7	19	8	5	1	8	19	7	4	0	6	16
79	L3PL	LDL-3 phospholipids	mg/dL	9	4	2	9	19	10	5	2	10	19	8	4	2	7	17
80	L4PL	LDL-4 phospholipids	mg/dL	8	5	0	7	21	9	5	0	9	17	8	5	0	6	20
81	L5PL	LDL-5 phospholipids	mg/dL	9	6	0	9	23	9	6	0	10	19	8	6	0	8	18
82	L6PL	LDL-6 phospholipids	mg/dL	12	6	0	11	29	13	7	2	13	27	12	6	2	11	23
83	L1AB	LDL-1 Apo-B100	mg/dL	15	7	5	13	28	15	7	6	13	31	14	6	5	14	26
84	L2AB	LDL-2 Apo-B100	mg/dL	8	5	0	7	19	8	5	2	8	19	7	5	0	7	16
85	L3AB	LDL-3 Apo-B100	mg/dL	11	5	4	10	20	11	5	4	11	20	10	4	4	10	19
86	L4AB	LDL-4 Apo-B100	mg/dL	10	6	0	9	25	11	5	0	11	20	10	7	0	9	25
87	L5AB	LDL-5 Apo-B100	mg/dL	13	8	0	13	31	13	8	0	14	27	12	8	0	13	29
88	L6AB	LDL-6 Apo-B100	mg/dL	18	10	0	16	43	19	11	4	18	40	18	10	3	16	38
89	H1TG	HDL-1 triglycerides	mg/dL	6	3	2	5	14	6	3	2	5	12	6	3	3	5	14
90	H2TG	HDL-2 triglycerides	mg/dL	3	1	1	3	7	3	1	1	3	5	3	1	2	3	7
91	H3TG	HDL-3 triglycerides	mg/dL	3	1	1	3	7	3	1	1	3	5	3	1	1	3	6
92	H4TG	HDL-4 triglycerides	mg/dL	3	1	1	3	6	3	1	1	3	4	3	1	1	3	6
93	H1CH	HDL-1 cholesterol	mg/dL	18	9	6	16	46	22	10	8	20	46	15	7	6	14	32
94	H2CH	HDL-2 cholesterol	mg/dL	9	3	4	8	15	10	3	5	9	15	8	2	4	7	15
95	H3CH	HDL-3 cholesterol	mg/dL	8	3	4	8	16	9	3	4	10	17	7	2	4	7	12
96	H4CH	HDL-4 cholesterol	mg/dL	10	7	0	9	24	12	7	0	13	25	9	6	0	8	21
97	H1FC	HDL-1 free cholesterol	mg/dL	4	3	0	4	11	6	3	0	5	11	3	2	0	3	9
98	H2FC	HDL-2 free cholesterol	mg/dL	2	1	1	2	4	3	1	1	3	4	2	1	1	2	4
99	H3FC	HDL-3 free cholesterol	mg/dL	2	1	0	2	5	2	1	0	2	5	2	1	0	1	4
100	H4FC	HDL-4 free cholesterol	mg/dL	3	2	0	3	8	3	2	0	3	8	2	2	0	2	6
101	H1PL	HDL-1 phospholipids	mg/dL	24	11	8	21	59	28	13	12	26	60	21	8	9	18	40
102	H2PL	HDL-2 phospholipids	mg/dL	15	4	8	14	25	16	5	8	15	25	14	4	9	14	22
103	H3PL	HDL-3 phospholipids	mg/dL	13	4	7	13	25	15	5	7	15	26	12	3	8	12	19
104	H4PL	HDL-4 phospholipids	mg/dL	15	8	0	14	32	17	9	3	20	34	13	7	0	14	28
105	H1A1	HDL-1 Apo-A1	mg/dL	27	17	0	21	78	33	21	4	32	80	22	13	3	18	48
106	H2A1	HDL-2 Apo-A1	mg/dL	18	6	8	16	33	19	7	9	18	34	16	5	10	15	27
107	H3A1	HDL-3 Apo-A1	mg/dL	23	7	11	22	44	25	8	13	25	44	22	6	12	21	36
108	H4A1	HDL-4 Apo-A1	mg/dL	46	20	11	44	89	53	22	12	55	92	42	17	15	40	81

	parameter			data of this study cohort (all samples)					COVID-19 patients					Non-COVID pneumonia controls				
	Lipoproteins																	
	abbreviation	full name	unit	mean	sd	$Q_{2.5\%}$	$Q_{50\%}$	$Q_{97.5\%}$	mean	sd	$Q_{2.5\%}$	$Q_{50\%}$	$Q_{97.5\%}$	mean	sd	$Q_{2.5\%}$	$Q_{50\%}$	$Q_{97.5\%}$
109	H1A2	HDL-1 Apo-A2	mg/dL	3	2	1	3	8	4	2	1	3	9	3	1	1	3	6
110	H2A2	HDL-2 Apo-A2	mg/dL	3	1	2	3	6	4	1	2	4	6	3	1	2	3	5
111	H3A2	HDL-3 Apo-A2	mg/dL	6	2	3	6	9	6	2	3	6	9	6	2	3	5	9
112	H4A2	HDL-4 Apo-A2	mg/dL	11	6	1	11	23	13	7	2	14	24	11	5	2	10	22
	Small Molecular Metabolites																	
113		Ethanol	mmol/L	0,17	0,29	0,00	0,00	0,99	0,29	0,41	0,00	0,19	1,20	0,09	0,13	0,00	0,00	0,40
114		Trimethylamine-N-oxide	mmol/L	0,00	0,02	0,00	0,00	0,09	0,01	0,03	0,00	0,00	0,10	0,00	0,01	0,00	0,00	0,00
115		2-Aminobutyric acid	mmol/L	0,03	0,05	0,00	0,00	0,16	0,02	0,04	0,00	0,00	0,12	0,04	0,06	0,00	0,00	0,16
116		Alanine	mmol/L	0,44	0,14	0,20	0,40	0,76	0,44	0,13	0,22	0,43	0,66	0,43	0,15	0,21	0,39	0,75
117		Asparagine	mmol/L	0,01	0,02	0,00	0,00	0,07	0,00	0,02	0,00	0,00	0,06	0,01	0,02	0,00	0,00	0,07
118		Creatine	mmol/L	0,06	0,09	0,00	0,02	0,44	0,05	0,10	0,00	0,02	0,32	0,06	0,08	0,00	0,03	0,21
119		Creatinine	mmol/L	0,12	0,05	0,00	0,11	0,28	0,12	0,06	0,05	0,11	0,27	0,12	0,05	0,05	0,12	0,24
120		Glutamic acid	mmol/L	0,23	0,16	0,00	0,19	0,86	0,20	0,10	0,08	0,18	0,46	0,25	0,19	0,09	0,19	0,82
121		Glutamine	mmol/L	0,63	0,22	0,00	0,64	1,01	0,67	0,21	0,23	0,69	1,05	0,60	0,22	0,00	0,62	0,94
122		Glycine	mmol/L	0,39	0,22	0,15	0,31	1,11	0,31	0,17	0,15	0,27	0,72	0,44	0,24	0,19	0,36	1,06
123		Histidine	mmol/L	0,08	0,02	0,04	0,08	0,14	0,08	0,02	0,04	0,08	0,13	0,08	0,02	0,04	0,08	0,13
124		Isoleucine	mmol/L	0,06	0,03	0,00	0,06	0,12	0,06	0,02	0,02	0,05	0,10	0,07	0,03	0,03	0,07	0,11
125		Leucine	mmol/L	0,11	0,04	0,05	0,10	0,22	0,09	0,03	0,04	0,10	0,15	0,12	0,04	0,05	0,10	0,22
126		Lysine	mmol/L	0,15	0,07	0,00	0,14	0,38	0,12	0,06	0,00	0,13	0,23	0,17	0,07	0,06	0,16	0,38
127		Methionine	mmol/L	0,06	0,03	0,00	0,07	0,11	0,05	0,04	0,00	0,06	0,10	0,07	0,03	0,00	0,07	0,11
128		N,N-Dimethylglycine	mmol/L	0,00	0,00	0,00	0,00	0,01	0,00	0,01	0,00	0,00	0,01	0,00	0,00	0,00	0,00	0,01
129		Ornithine	mmol/L	0,03	0,05	0,00	0,00	0,16	0,02	0,04	0,00	0,00	0,14	0,03	0,05	0,00	0,00	0,15
130		Phenylalanine	mmol/L	0,12	0,05	0,05	0,11	0,22	0,10	0,03	0,05	0,11	0,14	0,14	0,05	0,06	0,14	0,22
131		Proline	mmol/L	0,36	0,32	0,00	0,45	1,10	0,35	0,32	0,00	0,44	0,78	0,37	0,33	0,00	0,46	1,07
132		Sarcosine	mmol/L	0,00	0,00	0,00	0,00	0,00	0,00	0,00	0,00	0,00	0,00	0,00	0,00	0,00	0,00	0,00
133		Threonine	mmol/L	0,03	0,07	0,00	0,00	0,26	0,04	0,10	0,00	0,00	0,29	0,02	0,04	0,00	0,00	0,13
134		Tyrosine	mmol/L	0,06	0,02	0,00	0,06	0,10	0,05	0,02	0,03	0,05	0,08	0,06	0,02	0,00	0,06	0,10
135		Valine	mmol/L	0,24	0,07	0,13	0,23	0,41	0,22	0,06	0,13	0,21	0,32	0,25	0,08	0,14	0,23	0,41
136		2-Hydroxybutyric acid	mmol/L	0,07	0,11	0,00	0,00	0,35	0,06	0,12	0,00	0,00	0,37	0,07	0,11	0,00	0,00	0,30
137		Acetic acid	mmol/L	0,04	0,02	0,01	0,03	0,14	0,03	0,03	0,01	0,03	0,09	0,04	0,02	0,01	0,03	0,10
138		Citric acid	mmol/L	0,09	0,06	0,00	0,08	0,24	0,09	0,07	0,00	0,07	0,23	0,09	0,07	0,00	0,08	0,23
139		Formic acid	mmol/L	0,06	0,02	0,03	0,06	0,12	0,06	0,02	0,04	0,06	0,10	0,06	0,02	0,03	0,06	0,11
140		Lactic acid	mmol/L	2,33	1,61	0,66	1,90	8,12	2,46	1,83	0,73	1,90	7,65	2,25	1,47	0,85	2,00	5,65
141		Succinic acid	mmol/L	0,01	0,03	0,00	0,00	0,10	0,01	0,02	0,00	0,00	0,05	0,02	0,03	0,00	0,00	0,10
142		Choline	mmol/L	0,00	0,01	0,00	0,00	0,05	0,00	0,02	0,00	0,00	0,05	0,00	0,01	0,00	0,00	0,05
143		2-Oxoglutaric acid	mmol/L	0,00	0,01	0,00	0,00	0,04	0,00	0,01	0,00	0,00	0,02	0,01	0,01	0,00	0,00	0,04
144		3-Hydroxybutyric acid	mmol/L	0,41	0,60	0,00	0,14	2,31	0,31	0,47	0,02	0,13	1,35	0,47	0,67	0,00	0,15	2,29
145		Acetoacetic acid	mmol/L	0,05	0,10	0,00	0,00	0,44	0,01	0,02	0,00	0,00	0,06	0,07	0,12	0,00	0,00	0,42
146		Acetone	mmol/L	0,06	0,09	0,00	0,03	0,41	0,05	0,06	0,00	0,03	0,19	0,07	0,10	0,00	0,03	0,40
147		Pyruvic acid	mmol/L	0,10	0,06	0,03	0,09	0,26	0,11	0,06	0,04	0,09	0,24	0,10	0,06	0,03	0,09	0,25
148		D-Galactose	mmol/L	0,00	0,00	0,00	0,00	0,00	0,00	0,00	0,00	0,00	0,00	0,00	0,00	0,00	0,00	0,00
149		Glucose	mmol/L	7,85	3,40	3,79	6,95	17,05	7,75	3,37	4,10	6,90	15,50	7,91	3,46	4,00	7,00	17,00
150		Glycerol	mmol/L	0,47	0,34	0,00	0,38	1,61	0,44	0,32	0,00	0,36	1,23	0,49	0,36	0,00	0,45	1,20
151		Dimethylsulfone	mmol/L	0,01	0,01	0,00	0,00	0,05	0,01	0,02	0,00	0,00	0,06	0,00	0,01	0,00	0,00	0,02
152	Ca-EDTA	Calcium Ethylenediaminetetraacetic acid	mmol/L	0,03	0,23	0,00	0,00	0,05	0,06	0,36	0,00	0,00	0,50	0,00	0,00	0,00	0,00	0,00
153	K-EDTA	Potassium Ethylenediaminetetraacetic acid	mmol/L	0,11	0,95	0,00	0,00	0,21	0,27	1,51	0,00	0,00	2,10	0,00	0,00	0,00	0,00	0,00
	Inflammatory Markers (PACS pannel)																	
154	GlycA	GlycA signal	p.d.u	1,13	0,24	0,76	1,09	1,69	1,08	0,26	0,77	1,07	1,60	1,16	0,22	0,77	1,18	1,52
155	GlycB	GlycB signal	p.d.u	0,45	0,09	0,31	0,45	0,64	0,43	0,10	0,31	0,43	0,64	0,46	0,08	0,31	0,47	0,60
156	Glyc	Glycoprotein composite signal	p.d.u	1,57	0,33	1,07	1,53	2,33	1,51	0,36	1,08	1,51	2,21	1,62	0,30	1,08	1,65	2,09
157	SPC	Supramolecular phospholipid composite	p.d.u	1,68	0,67	0,74	1,53	3,49	1,99	0,74	0,85	1,85	3,62	1,48	0,53	0,85	1,40	2,96
158	Glyc/SPC	Glyc/SPC ratio	p.d.u/p.d.u	1,11	0,56	0,38	0,97	2,40	0,91	0,55	0,38	0,77	2,46	1,25	0,53	0,39	1,25	2,34

p.d.u procedure defined units
